# Incidence and management of early postoperative complications in lamellar corneal transplantation

**DOI:** 10.1007/s00417-023-06073-6

**Published:** 2023-04-27

**Authors:** Davide Romano, Francesco Aiello, Mohit Parekh, Hannah J. Levis, Kunal A. Gadhvi, Antonio Moramarco, Pietro Viola, Luigi Fontana, Francesco Semeraro, Vito Romano

**Affiliations:** 1 Ophthalmology Department, University Hospitals of Leicester NHS Trust, Leicester, UK; 2 Eye Clinic, ASST Spedali Civili Di Bescia, Department of Medical and Surgical Specialties, Radiological Sciences, and Public Health, University of Brescia Medical School, Piazzale Spedali Civili, 1, 25125 Brescia, Italy; 3 Ophthalmology Unit, Department of Experimental Medicine, University of Rome “Tor Vergata”, Rome, Italy; 4 Department of Ophthalmology, Schepens Eye Research Institute, Massachusetts Eye and Ear, Harvard Medical School, Boston, MA USA; 5 Department of Eye and Vision Science, Institute of Life Course and Medical Sciences, University of Liverpool, Liverpool, UK; 6 Department of Corneal Diseases, St. Paul’s Eye Unit, Royal Liverpool University Hospital, Liverpool, UK; 7 Ophthalmology Unit, IRCCS Azienda Ospedaliero-Universitaria Di Bologna, Bologna, Italy; 8 Department of Ophthalmology, San Bartolo Hospital, Vicenza, Italy

**Keywords:** Corneal transplant, Complications, Penetrating keratoplasty, Deep anterior lamellar keratoplasty, Descemet stripping automated endothelial keratoplasty, Descemet membrane endothelial keratoplasty

## Abstract

**Purpose:**

To provide a comprehensive review of the incidence, risk factors, and management of early complications after deep anterior lamellar keratoplasty (DALK), Descemet stripping automated keratoplasty (DSAEK), and Descemet membrane endothelial keratoplasty (DMEK).

**Methods:**

A literature review of complications, that can occur from the time of the transplant up to 1 month after the transplant procedure, was conducted. Case reports and case series were included in the review.

**Results:**

Complications in the earliest postoperative days following anterior and posterior lamellar keratoplasty have shown to affect graft survival. These complications include, but are not limited to, double anterior chamber, sclerokeratitis endothelial graft detachment, acute glaucoma, fluid misdirection syndrome, donor-transmitted and recurrent infection, and Uretts-Zavalia syndrome.

**Conclusion:**

It is essential for surgeons and clinicians to not only be aware of these complications but also know how to manage them to minimize their impact on long-term transplant survival and visual outcomes. 
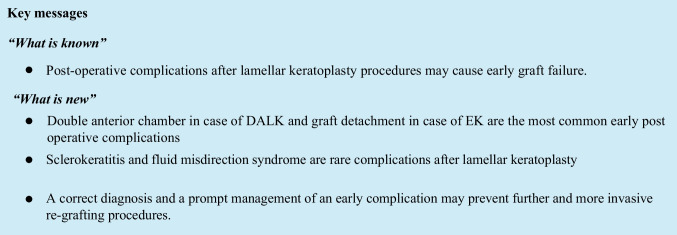

## Introduction

Over the last two decades, significant advances have been made in corneal transplantation techniques. Surgical management of corneal disorders has evolved from the replacement of full thickness cornea to replacing only the diseased corneal stroma or endothelium [1, 2]. This shift from penetrating keratoplasty (PK) to selective transplants like anterior and posterior lamellar surgery has been justified by earlier visual rehabilitation, maintenance of the structural integrity of the eye, and reduced incidence of allograft rejection [1–3]. Posterior lamellar (endothelial) keratoplasty techniques include Descemet stripping automated endothelial keratoplasty (DSAEK), in which posterior donor stroma is transplanted with endothelium, or Descemet membrane endothelial keratoplasty (DMEK), in which only Descemet membrane and endothelium are transplanted. Both, DSAEK and DMEK, compared to PK, have the advantage of better postoperative vision, in view of lack of suture induced astigmatism, and lower risk of rejection [4, 5]. According to the Eye Bank Association of America, the number of PKs and anterior lamellar keratoplasty (ALK) undertaken declined from 21,970 to 17,409 (− 20%) and from 1041 to 745 (− 28%) respectively between 2010 and 2019. However, during the same period, an increase from 19,159 to 30,650 (60%) in endothelial keratoplasty (EK) was observed [6].

Comparing the possible complications after PK with those after lamellar keratoplasty, some of the complications related to sutures, such as ametropia, and late wound dehiscence, are obviated only in EK and not in ALK. However, lamellar keratoplasties are not free from complications which could affect graft survival. These complications occur in the earliest weeks post-transplant and in most cases are evident at the first postoperative clinic examination. The diagnosis and successful management of these early complications is crucial for long-term lamellar graft survival [7]. In this review, we highlight diagnosis, management, and treatment strategies for early complications of lamellar keratoplasty seen in the first postoperative month.

## Methods

A single investigator (DR) used the MEDLINE database (via PubMed) to search for and identify articles for inclusion in this review. Keywords used were “Complication” AND “ALK *OR* DALK *OR* EK *OR* DMEK *OR* DSAEK.” Articles, including case reports and case series, up to June 2022 were included. Postoperative complications which can manifest up to 4 weeks post-transplant were included.

Complications were divided into anterior lamellar keratoplasty complications (double anterior chamber, sclerokeratitis), endothelial keratoplasty complications (endothelial graft detachment, inverted graft, and acute glaucoma), and anterior lamellar keratoplasty and endothelial keratoplasty complications (donor-transmitted and recurrent infection and Uretts-Zavalia syndrome). Table 1 summarizes the complications included in this review.

**Table 1:** List of the early complications which may occur up to 4 weeks after anterior lamellar keratoplasty (ALK), endothelial keratoplasty (EK), and common in both

ALK complications	EK complications	AK and EK complications
Double anterior chamber	Endothelial graft detachment	Donor-transmitted and recurrent infection
Sclerokeratitis	Inverted graft	Urretts-Zavalia syndrome
	Acute glaucoma	
	Fluid misdirection syndrome	

## Anterior lamellar keratoplasty complications

### Double anterior chamber

#### Definition, epidemiology, and risk factors

Double anterior chamber describes the separation of host Descemet membrane (DM) from the donor stroma. It occurs in about 10% of cases after ALK, with no difference accordingly to surgeon grade, and it has been found that 0.4% are converted to PK [2, 8, 9].

It most frequently occurs following central (within the central 4 mm), rather than midperipheral (within 4–6 mm from the center), Descemet membrane perforation. It is either apparent during surgery or not, with subsequent continuous flow of aqueous between both sides of the Descemet Membrane, and after a type 2 bubble [10].

Intraoperative complications are not the only causes of double chamber, as it may occur in cases of mismatch between donor-recipient curvature and presence of host risk factors, such as pseudophakia, and stromal corneal scarring in either eyes with or without keratoconus [10, 11].

When considering if there are differences in double chamber formation risk following manual, pneumatic, or viscoelastic dissection, comparative studies are lacking, whereas there is evidence to suggest that femtosecond-assisted deep anterior lamellar keratoplasty (DALK) is associated with a lower risk of intraoperative Descemet membrane perforation and double chamber formation, compared to manual dissection [12].

#### Management

If micro perforation occurs during surgery, it is not advisable to perform a Descemet-On DALK, which consists of transplanting a full thickness donor graft with intact DM and endothelium, because the double chamber may persist if the endothelium has not been removed from the donor [13, 14].

Postoperative management depends on the area of detachment. Small, peripheral areas of detachment that do not extend can be observed without intervention as most naturally resolve in a few days or cause no visual symptoms in the long term. However, in most cases, the area of detachment is large, central, or symptomatic and in these cases air or gas tamponade is recommended, with a high chance of reattachment after just one rebubbling [10]. In cases of Descemet membrane detachment (DMD) requiring rebubbling, it is advisable to not delay, because the DM will become fibrotic and the chance of successful rebubbling will be reduced [15, 16]. However, to date, it is not possible to define a precise deadline to perform rebubbling, in view of the lack of prospective randomized control studies, although some authors suggest not waiting more than 1 month in cases of DMD [17, 18].

The aim of injecting air or gas tamponade is to drain the fluid at graft-host junction possibly sealing the micro perforation. Intracameral injection of air, sulfur hexafluoride (SF_6_), or perfluoropropane (C_3_F_8_) should be combined with either pupil dilation or peripheral iridotomy (PI) and intravenous mannitol 20% to prevent pupil block glaucoma [19, 20].

In cases of anterior chamber (AC) reformation, it has been demonstrated that the average loss of endothelial cells is greater than 20% [21], while in cases of unsuccessful rebubbling and subsequent persistence of double anterior chamber, a second keratoplasty may be necessary.

Besides PK or EK [16], other rescuing techniques have been proposed. The first employs the use of anchoring sutures, which require filling the AC with air and then placing 3 or 4 sutures at the same interval degree (90–120°) in the deep stroma, piercing through the limbus and penetrating the detached Descemet membrane and donor cornea in an uppercut fashion [22].

Additionally, the use of an amniotic membrane (AM) patch has been reported to seal the DM rupture [23]. This procedure requires removal of all the graft sutures, lifting of the graft and placing in a saline container. Air subsequently is injected in the AC and, using a sponge, the DM is dried. A patch of cryopreserved acellular AM is fixed over the perforation site with a small amount of fibrin glue and then the same graft must be resutured using 10–0 nylon interrupted sutures.

Another option may be injecting an air bubble in the anterior chamber and then draining it out using a spatula as an iris repositor thorough a partial thickness corneal tunnel [16, 24].

Apart from the cases previously mentioned above, three cases of spontaneous resolution of double anterior chamber in 6–8 weeks previously unresponsive to multiple attempts of rebubbling have been reported. Interestingly, they were managed by the “wait and see” [25, 26] approach or therapeutically by adding topical hypertonic eyedrops [27].

Along with traumatic DM tear, a case of spontaneous DM tear has been reported in the literature, with no subsequent DMD, 4 weeks following an uncomplicated big-bubble DALK [28]. Possible reasons for spontaneous DM tears may be due to stretching and bending of the DM following deep and tight stromal suture and the progressive resolution of the corneal stromal oedema in the first month after surgery. Indeed, the resolution of cornea oedema may cause a further stretching of the posterior corneal surface and an increase in tension along DM [28].

Clinically, the patient presented with circumscribed eccentric stromal oedema, and the management was conservative, with only topical use of steroid eye drops, with a partial resolution of oedema after 3 months. DMD did not occur in the follow-up time [28].

### Sclerokeratitis

#### Definition, epidemiology, and risk factors

Post-keratoplasty atopic sclerokeratitis is a strong inflammatory reaction that simulates a graft rejection [29]. It is a rare complication, reported only in a few case series of PK and DALK, and occurs in the early days after the transplant (1 to 4 weeks) [29–32]. If not diagnosed and treated in time, it can cause early graft rejection [29].

**Fig. 1:**
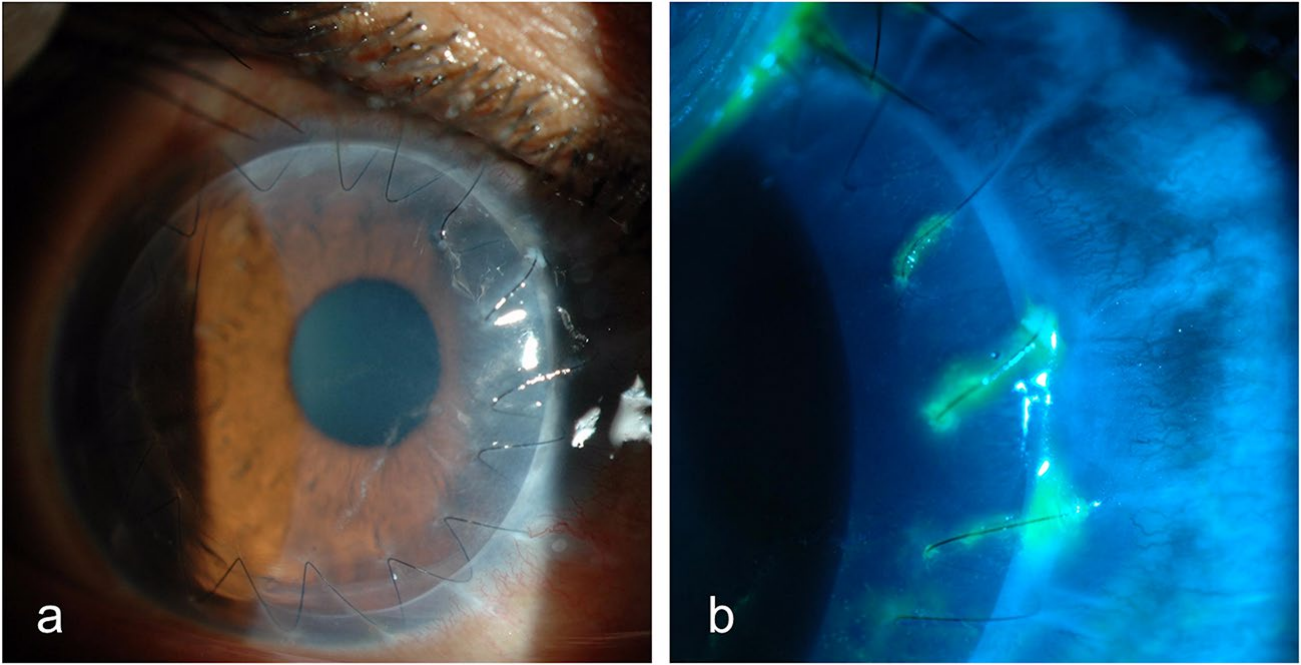
Sclerokeratitis after DALK. **a** Loose suture with injected neovessels. **b** Suture mucus collection highlighted with fluorescein staining and blue light

Signs are severe diffuse inflammation of the sclera, loosening of the sutures (Fig. 1), and persistent epithelial defects, while pain, photophobia, and epiphora are among the most common symptoms reported by patients [29–31].

Risk factors are active and not well-controlled atopic diseases (dermatitis, asthma, atopic keratoconjunctivits, elevated levels of serum immunoglobulin IgE), blepharitis, and corneal neovascularization [29–32].

#### Management

The management requires a rapid surgical approach, made by removal of the loose sutures and re-suturing with interrupted sutures if previously running sutures were used, as sclerokeratits tends to be unresponsive to topical corticosteroid therapy alone [29–32]. After the resuturing, it is advisable to restart immunosuppression until the inflammatory symptoms have subsided, which may take from 2 weeks up to 20 months [29–32]. The type of immunosuppression differs according to the literature. Along with using only topical 0.1% betamethasone four times per day [30], a combination of topical and systemic steroids (betamethasone 1 mg daily [29] or prednisolone 60–80 mg daily [31, 32]) and systemic cyclosporine (375 mg daily [29] or 7.5 mg/kg daily [31]) has been proposed. Systemic corticosteroids and cyclosporine should be tapered once the inflammation has subsided [29, 31, 32].

The efficacy of adding the cyclosporine to the systemic therapy is based on its ability to better inhibit interleukin-5 (IL-5), compared to corticosteroids [33]. Higher serum levels of IL-5 are present in patients with atopy, which, as reported before, are at higher risk of developing sclerokeratits [33].

However, prophylactic use of systemic cyclosporine in patients at risk is not recommended in view of possible systemic adverse events (i.e., nephrotoxicity, hepatotoxicity, infections, lymphoma, hirsutism, gingivitis, and central nervous system toxicity) [32, 34].

## Endothelial keratoplasty

### Endothelial graft detachment

#### Definition, epidemiology, and risk factors

Based on the presence or not of contact area between the donor tissue and the recipient bed, the detachment can be defined as partial (or incomplete) or total (complete) where the graft floats free in the anterior chamber [35].

The incidence of graft detachment differs according to the type of EK. It has been found to be higher in cases of DMEK compared to DSAEK [3, 35].

Reported rates of graft dislocation post-DSAEK vary widely from 0 to 42%, whereas in cases of DMEK, partial graft detachment has a reported incidence of 4–95% and a rebubbling rate of 2.4–82% (mean incidence 28.8%), while total graft detachment has an incidence of about 0.73–7% [35].

Risk factors associated with higher risk of postoperative graft detachment in cases of endothelial keratoplasty are graft preparation, learning curve, bullous keratopathy, graft size, age (younger recipient and older donor), previous PK and EK, presence of glaucoma surgery (both glaucoma drainage device and filtrating surgery), hypotony, abnormal anterior segment anatomy (peripheral synechiae, microphthalmos, aniridia), and incomplete descemethorexis [35–49].

The use of pre-loaded grafts, both in DSAEK and DMEK, is now an option [50, 51]. Non-statistically significant differences have been reported between preloaded and surgeon-prepared DSAEK graft, although the surgeon-prepared grafts have a higher adhesion force [52–54]. Instead, in cases of pre-loaded DMEK (pl-DMEK) versus pre-loaded ultrathin-DSAEK (pl-UT-DSAEK), pl-DMEK have higher rates of detachment compared to pl-UT-DSAEK [55].

Considering the lens status of the host, aphakia and anterior chamber intraocular lens are risk factors for graft detachment in cases of DMEK [56, 57], but not in cases of DSAEK [36].

Focusing instead on whether or not the type of AC tamponade may influence the graft detachment, in DMEK, SF_6_ 20% is associated with lower rate of graft detachment compared with 100% air [58, 59].

**Fig. 2:**
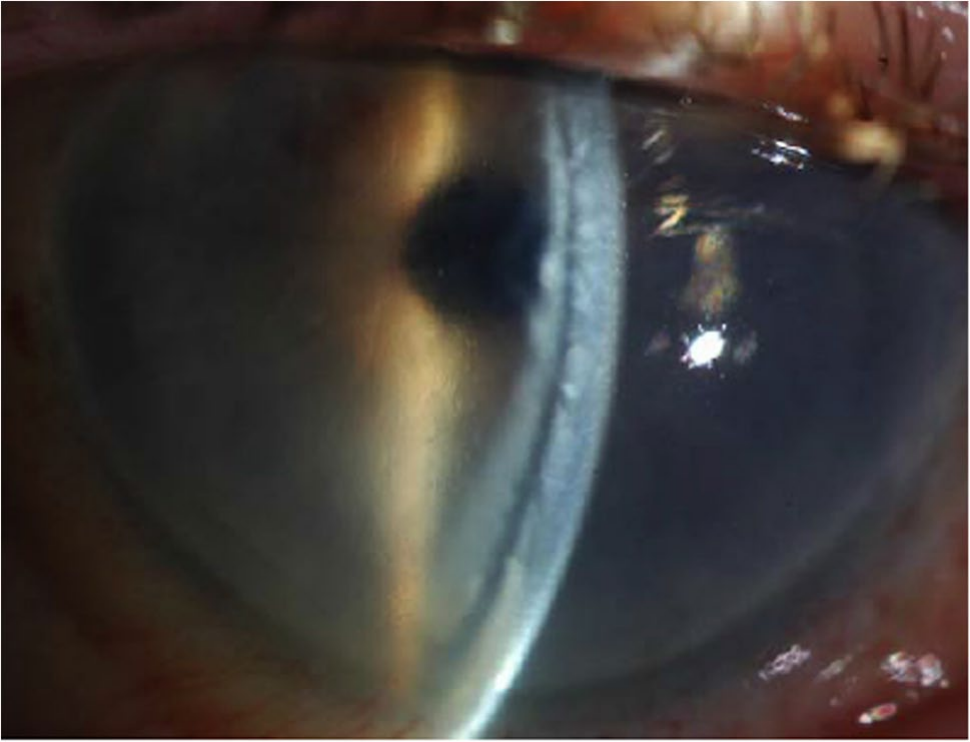
Slit lamp image of DSAEK graft detachment

**Fig. 3:**
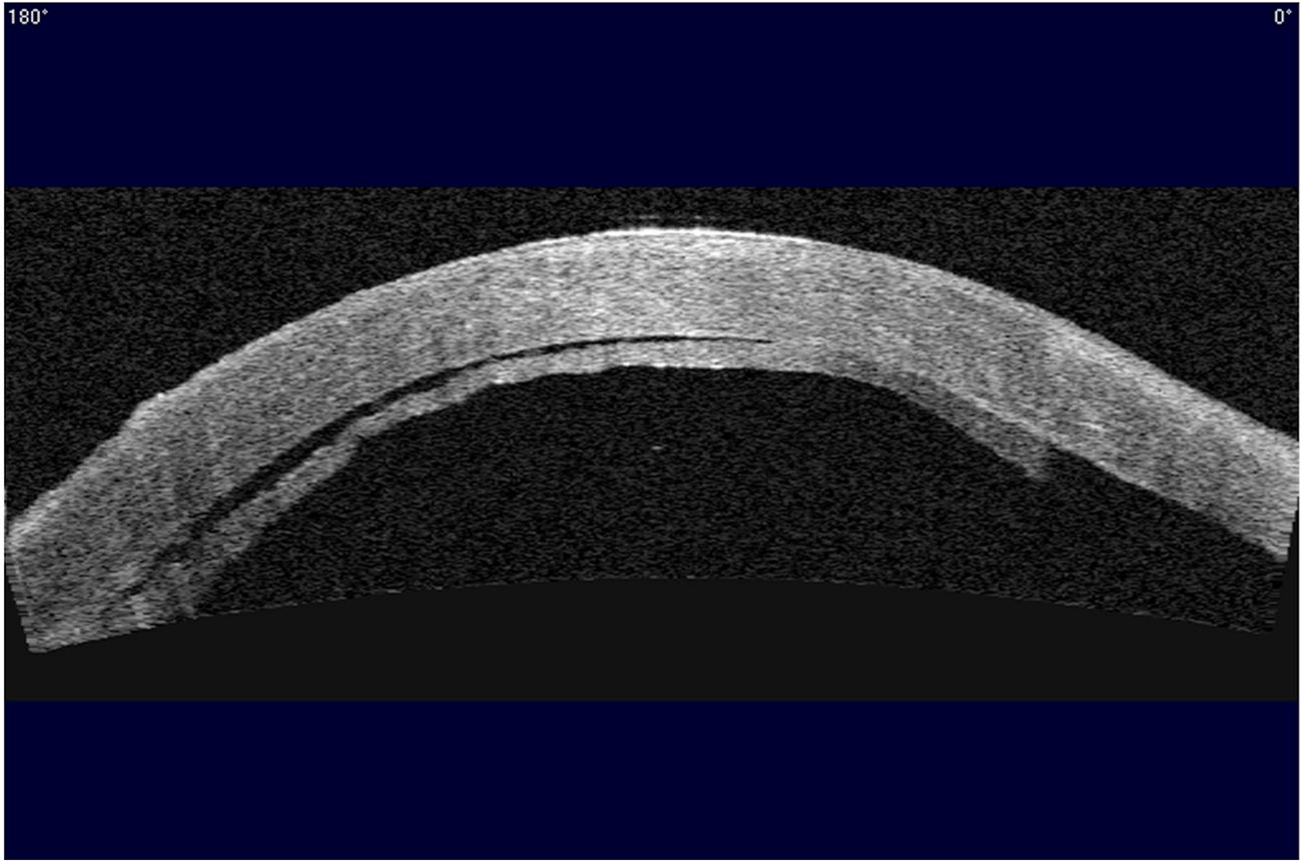
Anterior segment OCT of detached DSAEK graft

**Fig. 4:**
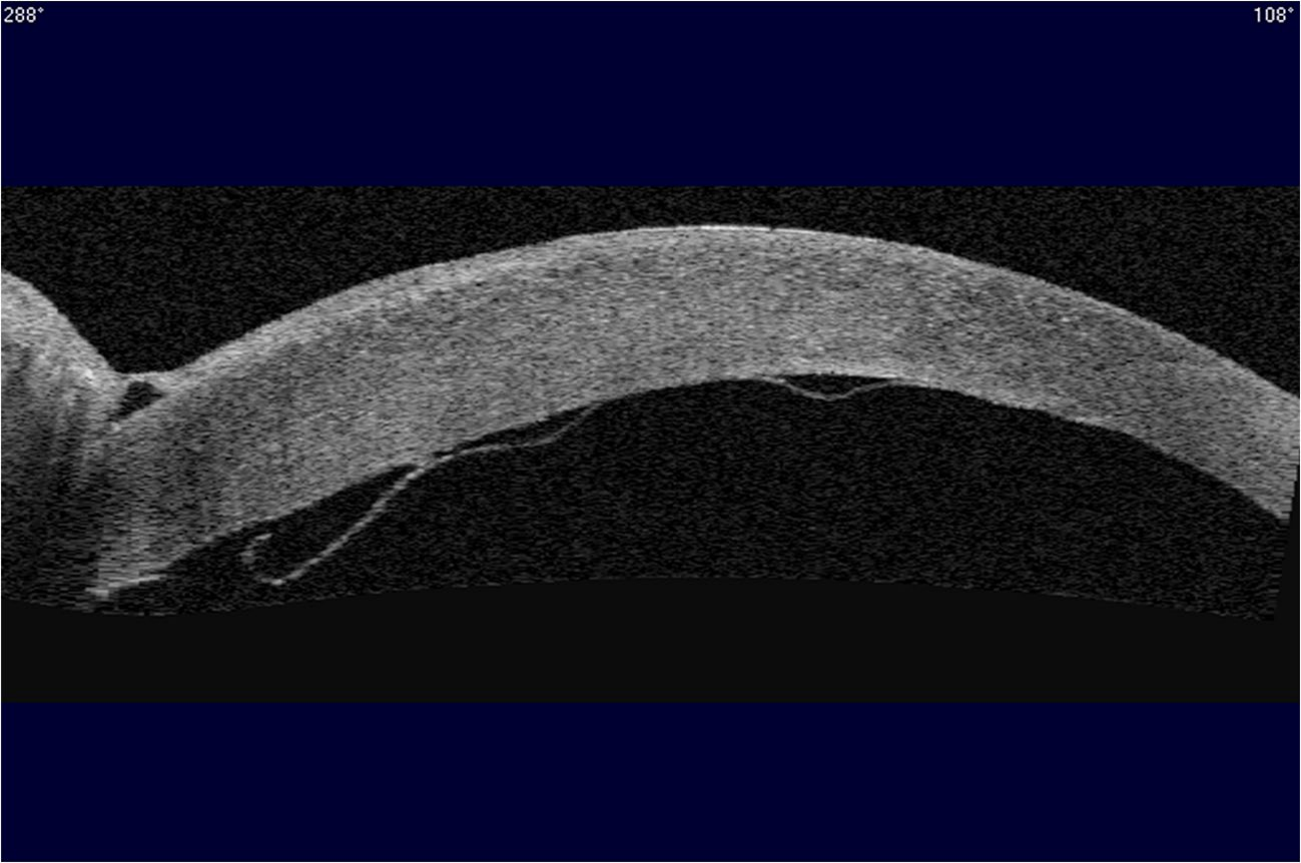
Anterior segment OCT of detached (9.5 mm) DMEK graft

Detachment post-DSAEK is diagnosed by slit lamp examination, usually clearly indicated by separation of the graft from the posterior recipient stroma (Fig. 2) but in some eyes it is indicated by an area of persistent corneal oedema. Diagnosis of detachment is more difficult following DMEK than DSAEK. The corneal stroma remains oedematous overlying the area of DMEK graft detachment, although it clears everywhere else [60, 61]. In some cases, the cornea may be too oedematous to clearly visualize graft position and it is difficult to determine if the graft is detached or corneal deturgescence is simply delayed [60–63]. Anterior segment optical coherence tomography (AS-OCT) and Scheimpflug imaging are helpful for immediate identification of graft detachment in such eyes post-DSAEK and post-DMEK (Figs. 3 and 4), particularly in eyes that have undergone recent surgery [64–69]. Comparing AS-OCT, Scheimpflug imaging, and slit-lamp biomicroscopy for the detection of DMEK graft detachment in the early postoperative phase, when the cornea is still oedematous, Moutsouris et al. reported that AS-OCT was superior to Scheimpflug imaging in confirming the diagnosis of graft attachment/detachment in 36% of eyes in which conclusive diagnosis could not be made by slit-lamp microscopy alone [66].

Identification and quantification of graft detachment is critically important post-DSAEK and DMEK as a decision must be made on the necessity for repeat bubble injection for graft repositioning and attachment [35, 70].

#### Management

In cases of DSAEK graft detachment, rebubbling should not be delayed, because even in cases of partial detachment, the risks are high that the area of the detachment will extend to the full graft. Only in cases of far-peripheral detachment rebubbling may not be an immediate choice, and a wait-and-see approach can be adopted.

In rebubbling, air or gas is injected into the anterior chamber posterior to the graft through a peripheral needle entry site and the graft positioned as during the initial surgical procedure. Use of a narrow gauge (27G or 30G) needle and relatively long intrastromal needle track reduces the problem of air reflux. This can be done at the slit lamp or on the operating table. Drainage of the pre-descemetic fluid via an external stab incision or internal aspiration with a needle may accompany the procedure for better reattachment. A longer period posturing supine usually allows secure attachment of the graft. Repositioning the DSAEK grafts by filling the anterior chamber with air is effective in managing dislocations, and some authors advocate the use of high-density gases for the repositioning of the graft (i.e., SF_6_ and C_3_F_8_) [71–73]. To date, no prospective or comparative studies on these management options for EK detachment have been published.

The management of graft detachment post-DMEK differs from DSAEK. The most common graft detachment following DMEK is a partial one, with a reported incidence of 4–95% and a rebubbling rate of 2.4–82% (mean incidence 28.8%), while total graft detachment has an incidence of about 0.73–7% [35, 74–78].

Various classifications of DMEK graft detachment based on OCT appearances have been proposed [79–81]. The key benefit of OCT is imaging the extent of detachment and its proximity to the visual axis, which cannot be clearly identified on slit lamp in many eyes, hence making it difficult to plan any subsequent air injection.

Fortunately, most post-DMEK detachments are non-progressive, and intervention is not required. The impact on the postoperative management of a given detachment depends on its size and location. However, numerous case reports on spontaneous resolution of corneal oedema, either by spontaneous reattachment of graft Descemet membrane [82–88] or migration of proximal endothelial cells, have been reported with excellent visual outcome [89, 90]. Detachments < 1/3rd of the graft surface area, non-scrolled in configuration, located at the periphery of the graft, and distant from the visual axis are usually asymptomatic and do not require rebubbling [63]. Usually all oedema resolves (typically within 3 months of surgery) resulting in good visual acuity. Assia et al. reported spontaneous reattachment of DMD within 2–3 months, whereas the mean period for spontaneous reattachment of DMD in another case series was 9.8 weeks (range 3–20 weeks) [60–63, 81].

Instead, detachments > 1/3rd of the graft surface area, scrolled in configuration, located in the center or involving the visual axis should be managed by repeat air/gas injection to reattach the graft. The rebubbling procedure should not be delayed, in order to hasten visual rehabilitation and to prevent the wrinkling, fibrosis, and shrinkage of graft Descemet membrane, which results in poor visual outcome [17].

There are various techniques for management of graft detachment. Sharma et al. use intracameral injection of 14% C_3_F_8_ except in cases of superior DMD, reporting successful management [91].

As mentioned before, the use of 20% SF_6_ as a tamponade is associated with 58% less rebubbling procedures compared with air [59]. However, a case series reported that 10% C_3_F_8_ is a better option as a tamponade in cases of previous failure observed with air- or SF_6_-assisted rebubbling attempts [92].

Regarding the toxicity of tamponade agents, animal studies indicate a similar endothelial toxicity profile for all three gases [93]. The overall efficacy of rebubbling is high, with a success of reattachment of the graft in 68–96.5% cases of DSAEK [94–96] and 79–92% in cases of DMEK [17, 70, 97].

Alternatively, if rebubbling is not performed, corneal clearance usually occurs but over a longer time period (6 months, on average) and only 50% of these eyes reach a visual acuity of 20/40 [60–63, 89]. As discussed in cases of DMD following microperforation in cases of DALK, rebubbling after 1 month is not always effective and the non-adherent segment may become fibrotic. Hence, it is recommended to perform a rebubbling if the graft is detached and the cornea does not clear within 1 month [17, 18, 35].

Effect of rebubbling on graft endothelial cell density (ECD) has been evaluated. In cases of single rebubbling versus no rebubbling, no differences in ECD are reported, while in cases of more than one rebubbling, a higher rate of endothelial cell loss is reported [98–101].

Re-transplantation is feasible, but may be considered as a last resort, since it is expensive, laborious, and risks further complications.

Injection technique varies between surgeons; it is usually performed at the slit lamp using a 27–30G needle or Fogla air injection cannula, mounted on a 3-ml syringe, through a new paracentesis to create an air-tight seal afterwards. The paracentesis can be made by a 20–23G side port blade and acts also as a valve allowing better control over intraocular pressure and percentage anterior chamber air fill. Attention should be paid in cases of DMEK rebubbling on a previous PK, as it has been reported in literature that it may causes wound dehiscence at the graft-host junction [102].

The site of paracentesis can be in the inferior temporal quadrant or in a site where Descemet membrane is still attached [17, 103]. OCT imaging of graft detachment is essential to plan the injection. Use of a speculum facilitates access and forcep fixation of the limbus stabilizes the globe and allows counter-pressure when inserting the needle. Adequate injection volume and posturing of sufficient duration to maintain bubble tamponade are more important compared to the injection of air or gas.

**Fig. 5:**
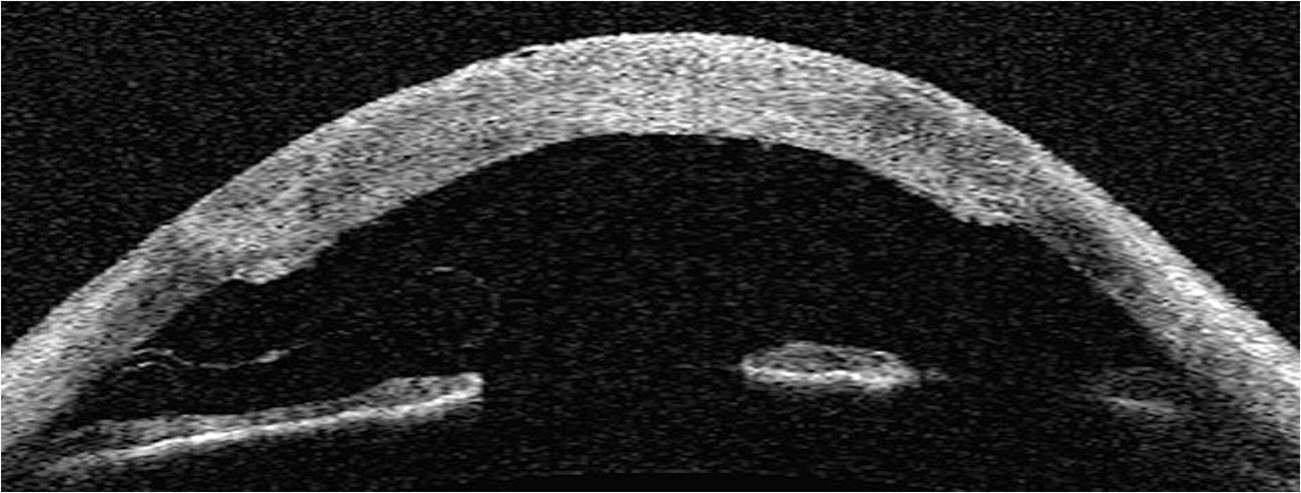
Anterior segment OCT of a fully detached DMEK graft lying on the iris plane

Instead, a complete detachment post-DMEK (Fig. 5) is more straightforward with respect to decision-making. In these cases, the graft entirely separates from the recipient posterior corneal surface, and it is usually free-floating in the anterior chamber. These grafts never re-attach and the entire cornea thus remains oedematous. In these cases, it may be necessary to remove and replace the graft, or alternatively, a rescue technique has been proposed [104].

This technique requires staining of the graft in the anterior chamber with trypan blue, making a 20-gauge paracentesis and immediately injecting air to avoid the staining of the host stroma. Subsequently, the stained DMEK graft is tapped to open it, and then attached to the host stroma injecting an air bubble.

The combined yield from AS-OCT at 1 h and 1 week post-operation has proven informative to predict if a detachment is likely to be transient or lasting [63]. At 1 week, if the graft is completely attached, then it should remain detachment-free. If any detachment is identified on the scan made at 1 h after surgery, the patient should be reviewed carefully at 1 week, and if this exam also shows the same detachment, then spontaneous re-attachment is unlikely, occurring in only 44% of cases. Conversely, if no detachment is present at 1 h postoperative, but is seen at week 1, then spontaneous re-attachment is likely, occurring in nearly 90% of cases [60–63]. Failure to separately enumerate graft detachment rates and graft rebubbling rates makes comparison of results in published reports difficult.

### Inverted grafts after endothelial keratoplasty

#### Definition and management

**Fig. 6:**
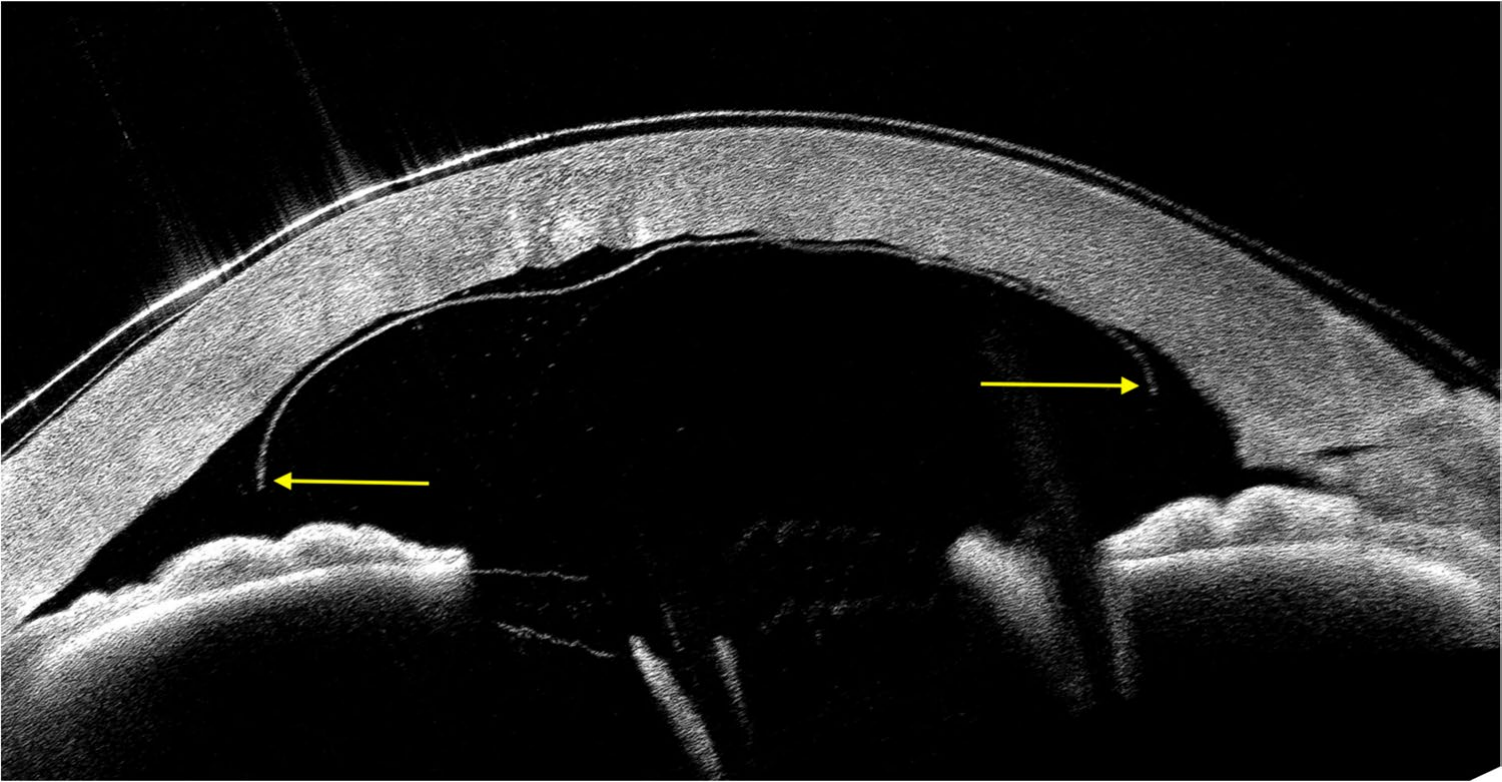
Anterior segment OCT of an inverted (upside-down) DMEK graft. Curled edges facing the anterior chamber are marked with yellow arrow at the extremity of the graft

In cases of intraoperative attachment of grafts in an inverted position (endothelial layer facing the host stroma; upside-down graft), there are two pathognomonic signs: (1) an extremity of the graft free-floating with posteriorly curled edges, visualized using AS-OCT (Fig. 6); and (2) reverse corneal clearance. The latter means that gradually the cornea spontaneously clears except where the graft is attached [61, 105]. In such cases, it would be advisable to detach the graft, possibly injecting balanced salt solution (BSS) from a side port, tap on the cornea to scroll it completely, unfold it, and then inject air or gas to reattach it [105].

### Acute glaucoma

#### Definition, epidemiology, and risk factors

**Fig. 7:**
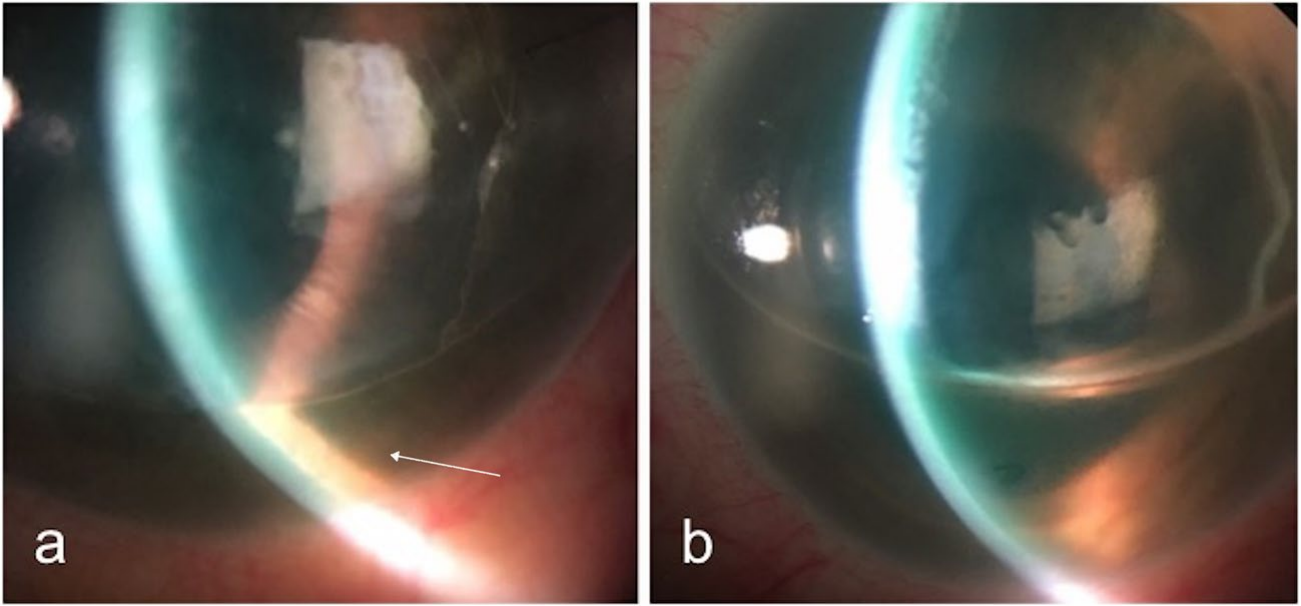
Pupil block after DMEK surgery. **a** Air bubble in the anterior chamber covering the whole pupil. The arrow shows the inferior iris pushed forward. **b** Resolution of pupil block after pupil was dilated

DSAEK and DMEK share the risk of postoperative acute glaucoma due to pupil block caused by the air or gas bubble in the anterior chamber (Fig. 7) or angle closure secondary to anterior iris dislocation caused by migration of the bubble to the posterior chamber. In a series of 13 out of 100 DSAEK eyes with IOP > 30 mmHg, 6 patients showed acute glaucoma due to posterior migration of air, and in 1, due to pupil block at day 1 post-op [106].

However, in a series of 40 eyes (24 DMEK and 16 DMEK rebubbled) treated with DMEK, all of them had increased IOP after surgery, despite the presence of inferior (PI) in all. Of these, 3 eyes (DMEK without rebubbling) had intraocular pressure (IOP) > 30 mmHg at 2 h after surgery with no pupillary block. None of the rebubbled eyes had IOP > 30 mmHg at 2 h. Overall, the IOP started to lower 3 h after surgery and IOP was normal in all patients at 1 week [107].

This opens up an interesting question of whether or not the early postoperative IOP ≥ 30 mmHg may affect the DMEK outcome pertaining to postoperative best-corrected visual acuity, central corneal thickness, and graft endothelial cell density. Temporary IOP elevation post-op did not seem to affect functional and morphological outcomes as reported by another retrospective analysis of 20 eyes from 172 patients with raised IOP in the first three postoperative days [108].

However, the acute pressure elevation can lead to vision loss due to glaucomatous optic neuropathy. The incidence of pupillary block glaucoma is between 0.1 and 9.5% [73, 109, 110]. Usually, an intraoperative inferior PI is performed to prevent this complication. Glaucoma remains a risk if the PI is not performed or if the anterior chamber bubble is so large that the iridotomy is itself blocked by the bubble. The risk of pupillary block is maximal for the first 24 h postoperatively, before the air bubble in the anterior chamber spontaneously reduces in size.

#### Management

In some cases of acute glaucoma, the attack can be reversed by pupil dilation, topical apraclonidine, systemic acetazolamide and supine positioning for 1–2 h, or evacuation of some air and replacement with saline [96]. If the IOP is elevated due to posterior migration of the air bubble, removal of air from the posterior chamber may be achieved using topical cycloplegic and mydriatic agent or using a needle to reduce pressure without the need for reformation of the anterior chamber drainage angle [96]. In cases of pupil block in which the air bubble appears to cover the inferior PI, a paracentesis should be performed to remove sufficient air to raise the aqueous humor meniscus above the PI. The air is released at slit lamp with a 30G needle on a 1-ml syringe.

A possible (rare) consequence of acute glaucoma due to pupillary block following endothelial keratoplasty is the Urrets-Zavalia syndrome that will be discussed in a dedicated paragraph.

### Fluid misdirection syndrome

#### Definition, risk factors, and management

Although rare, another condition should be ruled out in cases of high IOP after surgery and fluid misdirection syndrome (FMS). To date, cases of FMS following endothelial transplant, both DSAEK and DMEK, are still limited to a case series of 11 eyes, but it is a condition which surgeons and clinicians should be aware of [111].

The main risk factor for FMS is small hyperopic eyes (mean axial length in the case series 21.7 mm) with a shallow anterior chamber (mean anterior chamber depth in the case series 2.4 mm). Previous cataract surgery does not prevent FMS after endothelial keratoplasty [111].

Clinical findings of FMS are shallowing of anterior chamber despite the presence of PI, IOP > 21 mmHg, and lack of presence of any possible cause of angle closure. The management of acute FMS, with onset immediately after surgery, requires a pars plana decompression. Instead in cases of chronic FMS, which can occur days or months after surgery, and is characterized by a progressive shallowing of the anterior chamber, a step-by-step approach is recommended, starting with topical hypentise eyedrops and ciclopegic agents, and then, YAG laser iridotomy with anterior hyaloidotomy and posterior capsulotomy. In case of no resolution, total pars plana vitrectomy combined with zonulectomy, iridectomy, and capsulectomy is recommended [111].

## Anterior lamellar keratoplasty and endothelial keratoplasty complications

### Donor-transmitted and recurrent infection

#### Definition, epidemiology, and risk factors

Microbial keratitis is a serious complication of any type of keratoplasty and is usually associated with a poor visual prognosis because of the difficulty of successful treatment without residual scarring.

The reported incidence of infectious keratitis following ALK is 1% [112, 113]; however, Sharma et al. reported a rate of 11.11% for all types of infectious keratitis among 135 total ALK procedures [114]. In EK procedures, an estimated incidence of fungal infection between 0.5 and 0.7% has been reported [115].

However, at present, there are limited studies documenting infectious keratitis after endothelial or anterior lamellar keratoplasty to adequately assess the outcomes [115, 116].

**Fig. 8:**
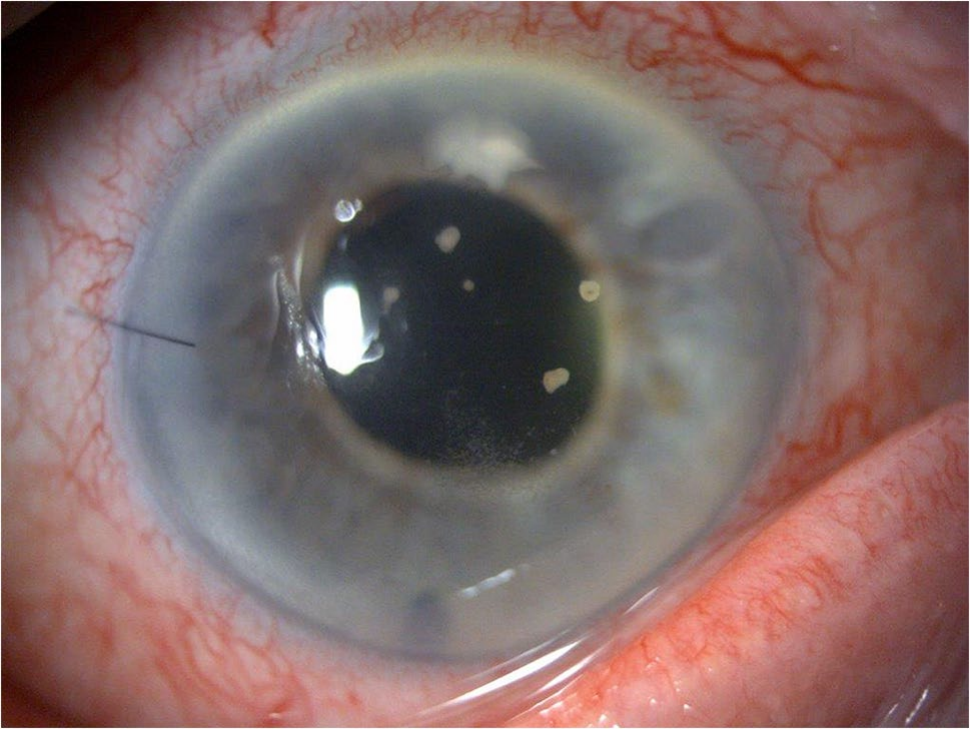
Candida interface infection 4 weeks after DMEK surgery

Following anterior or posterior lamellar keratoplasty, the features are white or creamy deposits at the interface between the host-donor (Fig. 8).

Candida is the most common reported pathogen and fungal etiology is suggested by an interval of 1–3 weeks post-transplant [117–128]. More than 85% of the cases of fungal keratitis recurrence occurred within 7 days of surgery [129].

In cases of de novo infection, the eye bank in which the donor cornea is processed must be informed and any available microbiology information on contamination of the donor scleral rim or culture media must be obtained. Indeed, according to the literature, up to 2.1% of corneal donor tissue is positive for fungal rim cultures, which may cause, if transplanted, a clinical infection in the host in about 5.6–13.5% cases [130–132].

Subsequently, pathogens isolated from eye bank samples are likely to correlate with in vivo infection in the recipient eye and provide a rational basis for therapeutic decisions until definitive information is available on isolates from the transplanted eye. All the available diagnostic methods (culture, smear, polymerase chain reaction testing, and confocal microscopy) must be used to confirm infection, isolate the pathogen, and inform the choice of anti-microbial agents.

Considering instead the risk of transmission of COVID-19 virus transmission from donor corneal tissues, a review from Saltz et al. reported no evidence of viable virus and no cases of transmission of SARS‑CoV‑2 [133].

#### Management

Reports on the efficacy of antimicrobial treatment in infectious keratitis after keratoplasty range from 43 to 74% [134]. Fungal and viral infections often require a combination of oral and topical therapy. Occasionally, it is simpler to remove an infected anterior lamellar graft because drug penetration is not efficient. Usually, a full-thickness transplant is performed or lamellar grafts in selected cases [135]. Evisceration is typically reserved for cases where infectious keratitis has progressed to severe endophthalmitis [134].

When the indication for lamellar graft is microbial keratitis in which a medical cure has not been achieved, the surgeon needs to be aware of the high risk of recurrence of infection after surgery. The identification of the pathogen (confirmed before surgery or using the excised tissue) will influence the postoperative treatment. If a microbiological diagnosis has not been obtained prior to surgery, it is mandatory to examine the excised corneal tissue by culture and other available methods. Until a confirmed microbiology diagnosis is available, antimicrobial therapy should be guided by pre-transplant clinical features and local epidemiology.

### Uretts-Zavalia syndrome

#### Definition, risk factors, and management

Uretts-Zavalia syndrome (UZS), also known as Castroviejo syndrome, is described as the appearance of a fixed and dilated pupil following intraocular surgery [136].

Initially reported following PK [136], it has also been described after ALK and EK [19, 137–141]. Its pathogenesis is still not clear, but it is likely that it is a consequence of acute glaucoma due to pupillary block, following DSAEK/DMEK, which leads to ischemia of the iris. However, it has also been reported under low postoperative IOP [141, 142].

Clinically, patients report pain in the hours following surgery. The usual finding at the first postoperative examination is a fixed dilated pupil, not reactive to light or accommodation and iris atrophy. Between 30 and 60% of patients with this complication will recover some form of pupil reactivity within 1 to 18 weeks, with some patients regaining normal pupil size. The prognosis depends on the severity of the iris ischemia/atrophy, and patients with marked atrophy of both the anterior and posterior layers of the iris will have long-term photophobia due to irreversible mydriasis and chronic low-grade iritis. Reports on treatment in the literature are anecdotal. In cases where there is iris-host cornea touch, separation using a cyclodialysis spatula has been suggested. In cases of elevated IOP due to blood or viscoelastic in the anterior chamber, a washout is recommended, which can be done using several techniques. The easiest is to perform two clear corneal incisions at 180° apart. Through one incision, BSS is irrigated, while the opposite incision is depressed to evacuate the blood. In cases of unsuccessful irrigation using BSS, or presence of large blood clots, irrigation and aspiration or anterior vitrectomy may be required [143]. An anterior chamber washout is recommended.
